# Association between low magnesium status and new-onset dementia in the general population: a propensity score-matched cohort study

**DOI:** 10.3389/fnut.2026.1886820

**Published:** 2026-06-08

**Authors:** Ying-Jen Chang, Chia-Li Kao, Kuo-Mao Lan

**Affiliations:** 1 Department of Anesthesiology, Chi Mei Medical Center, Tainan City, Taiwan; 2 Department of Recreation and Health-Care Management, College of Recreation and Health Management, Chia Nan University of Pharmacy and Science, Tainan City, Taiwan; 3 Department of Anesthesiology, E-Da Hospital, I-Shou University, Kaohsiung City, Taiwan; 4 Department of Anesthesiology, Chi Mei Hospital, Liouying, Tainan City, Taiwan

**Keywords:** cohort study, dementia, electronic health records, magnesium deficiency, propensity score matching, vascular dementia

## Abstract

**Background:**

Low magnesium status has been linked to cerebrovascular conditions that overlap with pathways implicated in dementia, yet prior studies have relied on single magnesium measurements obtained in earlier population-based cohorts. Whether repeatedly documented low magnesium status is associated with incident dementia in real-world clinical populations is unclear.

**Methods:**

This retrospective propensity score-matched cohort study used data from the TriNetX Global Collaborative Network (2016–2023). Adults aged ≥50 years with two serum magnesium values <1.7 mg/dL within a 1-year window were compared with matched controls with two normal values (1.70–2.20 mg/dL). A 1-year landmark period was applied before the outcome ascertainment. The primary outcome was incident all-cause dementia. The secondary outcomes included dementia subtypes, stroke, and mortality. Prespecified positive and negative control outcomes, subgroup and sensitivity analyses, sequential multivariable Cox regression, and an exploratory analysis of high magnesium status were conducted.

**Results:**

After propensity score matching, 162,936 patients were included in each group. Low magnesium status was associated with incident all-cause dementia (HR 1.33; 95% CI, 1.26–1.40, *p* < 0.001), with the strongest association observed for vascular dementia (HR 1.63; 95% CI, 1.42–1.88, *p* < 0.001). Associations were also observed for stroke (HR 1.37, *p* < 0.001) and mortality (HR 1.52, *p* < 0.001). The results remained consistent across the sensitivity and multivariable analyses. High magnesium status was associated with all-cause dementia (HR 1.25, *p* < 0.001) and vascular dementia (HR 1.40, *p* < 0.001), but not Alzheimer’s disease.

**Conclusion:**

Repeatedly documented low magnesium status was associated with an increased risk of incident all-cause dementia, particularly vascular dementia, suggesting a potential role of magnesium homeostasis in long-term cognitive function.

## 1 Introduction

Dementia is a leading cause of disability and loss of independence in the aging population (1). Beyond established risk factors such as age, vascular disease, diabetes, and neuropsychiatric comorbidities, increasing attention has been focused on metabolic and nutritional biomarkers that may capture systemic vulnerability before the onset of dementia (2, 3). Identifying clinically measurable factors associated with long-term cognitive risk may help define at-risk populations and inform preventive strategies for cognitive decline. Magnesium is an essential cation involved in neuronal excitability, synaptic transmission, vascular tone, glucose metabolism, and regulation of inflammation (4–9). Low magnesium status has been linked to cardiometabolic diseases, endothelial dysfunction, hypertension, stroke, and mortality, conditions that overlap with pathways implicated in dementia development (10). However, the epidemiological relationship between circulating magnesium levels and dementia remains unclear.

Several cohort studies have examined this association (11, 12). In the Rotterdam Study, both low and high serum magnesium levels were associated with an increased risk of dementia (13). In the Atherosclerosis Risk in Communities Neurocognitive Study (ARIC-NCS) cohort, low midlife serum magnesium levels were associated with incident dementia during long-term follow-up (14–16). The Copenhagen General Population Study similarly reported an association between extreme plasma magnesium concentrations and vascular-related non-Alzheimer’s dementia (17). Despite these contributions, there are still significant gaps. First, prior studies largely relied on a single magnesium measurement, which may reflect transient electrolyte shifts rather than a sustained magnesium status. Second, most available evidence is derived from cohorts with baseline measurements obtained in the 1990s or the early 2000s, whereas contemporary real-world data from recent electronic health records remain limited. Third, observational dementia studies are vulnerable to reverse causation, as prodromal cognitive decline may coincide with changes in nutrition, comorbidity burden, and laboratory tests (18, 19). Evidence incorporating repeated magnesium measurements, temporal separation from outcome ascertainment, dementia subtype analyses, and internal control outcomes is limited.

To address these gaps, we conducted a propensity score–matched cohort study using a large electronic health record network from 2016 to 2023 to evaluate whether repeatedly low magnesium levels were associated with incident dementia in adults aged ≥ 50 years.

## 2 Methods

### 2.1 Data source and ethical statement

This retrospective cohort study was conducted using TriNetX, a federated electronic health record research platform that provides access to longitudinal, de-identified clinical data from participating healthcare organizations through the Global Collaborative Network (20). As no directly identifiable information was accessible to the investigators, informed consent was waived. The study protocol was reviewed and approved by the Institutional Review Board of Chi Mei Medical Center, Tainan, Taiwan. This study was conducted in accordance with the Declaration of Helsinki and relevant institutional requirements.

### 2.2 Exposure definition

Adults aged ≥ 50 years were eligible if they had at least two serum or plasma magnesium measurements recorded between January 1, 2016, and December 31, 2023. To reduce misclassification caused by transient laboratory abnormalities, the magnesium status was defined using repeated measurements. Patients were assigned to the low magnesium status group if they had two documented magnesium values <1.7 mg/dL within a 1-year assessment window (21). The reference group included patients with two magnesium values within the normal range of 1.70–2.20 mg/dL during the same assessment period (21). The magnesium categories were defined *a priori* according to the predefined reference interval used in prior literature (21): low magnesium status was defined as <1.70 mg/dL, normal magnesium status as 1.70–2.20 mg/dL, and high magnesium status as >2.20 mg/dL. Because TriNetX does not provide site-specific laboratory reference ranges, magnesium status was classified using this predetermined interval. For both groups, the date of the second qualifying magnesium measurement was defined as the index date, ensuring that cohort entry occurred only after the repeated magnesium status had been established.

### 2.3 Exclusion criteria

Several exclusion criteria were applied to minimize confounding factors from acute illness, severe systemic disease, or pre-existing neurological disorders. Patients were excluded if they had acute kidney failure, sepsis, severe sepsis, or required critical care services within 1 month before the qualifying magnesium measurement, because these conditions may cause acute electrolyte changes. Patients with dementia or major neurodegenerative diseases before the index period were also excluded, including Alzheimer’s disease, vascular dementia, dementia in other diseases classified elsewhere, unspecified dementia, and other degenerative diseases of the nervous system. Additional exclusion criteria included stage 4–5 chronic kidney disease, end-stage renal disease, dialysis dependence, bipolar disorder, schizophrenia-spectrum or other psychotic disorders, Parkinson’s disease, bariatric surgery status or procedures, prior stroke, non-traumatic intracranial hemorrhage or intracranial injury, central nervous system neoplasms, and documented COVID-19 infection. These exclusions were intended to reduce confounding factors associated with abnormal magnesium levels, cognitive decline, or differential outcome ascertainment. The patient selection process is summarized in Supplemental Figure S1.

### 2.4 Landmark analysis

A 1-year landmark design was used to strengthen the temporal separation between magnesium status classification and subsequent dementia ascertainment. Patients with any dementia diagnosis before the landmark time point were excluded, including those with dementia recorded before or within 365 days after the index date. Patients were also required to remain alive during the first year after the index date of the study. Follow-up for incident dementia began 365 days after the index date and continued until the first occurrence of the outcome, censoring, or the end of the observation window.

### 2.5 Data collection and propensity score matching

Baseline covariates were extracted from the TriNetX database prior to the outcome analysis. To improve the comparability between patients with low magnesium status and those with normal magnesium status, 1:1 propensity score matching was performed. Propensity scores were estimated using prespecified covariates selected based on their clinical relevance to magnesium homeostasis and dementia risk. These included demographic characteristics, cardiometabolic and vascular comorbidities, renal and hepatic diseases, neuropsychiatric disorders, nutritional and inflammatory conditions, medication exposure, and available laboratory indicators related to kidney function, inflammation, nutritional status, glucose metabolism, hematologic status, and body composition. The complete list of covariates is provided in Supplemental Table S1.

Patients were matched using a greedy nearest-neighbor algorithm with a caliper width of 0.1 pooled standard deviation of the propensity score. Covariate balance was evaluated using standardized mean differences, with values of <0.10 considered acceptable. Propensity score density plots were inspected before and after matching to evaluate cohort overlap and the adequacy of the matching process.

### 2.6 Primary, secondary, and control outcomes

The primary outcome was incident all-cause dementia, defined by a newly recorded International Classification of Diseases, Tenth Revision, Clinical Modification (ICD-10-CM) code for Alzheimer’s disease (G30), vascular dementia (F01), dementia in other diseases classified elsewhere (F02), or unspecified dementia (F03). Patients with any of these dementia diagnoses before or within the 1-year landmark period were excluded to support incident case ascertainment. Outcome ascertainment began after the completion of the 1-year landmark period and extended from 365 to 3,650 days after the index date.

Secondary outcomes included dementia subtypes, classified as Alzheimer’s disease (G30), vascular dementia (F01), and other or unspecified dementia (F02 or F03), as well as stroke (I63) and all-cause mortality. Dementia subtypes were classified according to the first recorded subtype-specific code during follow-up. For each outcome, follow-up continued until the first recorded event, censoring at the last available clinical record, death (when applicable), or completion of the observation window.

Prespecified control outcomes were used to assess the internal consistency of the study design. Hypokalemia was selected as a positive control because magnesium depletion can impair renal potassium conservation and is clinically linked to potassium abnormalities. Hypokalemia was defined by either a diagnostic code for hypokalemia or a serum potassium concentration ≤3.5 mmol/L. Acute appendicitis, defined using the ICD-10-CM code K35, was selected as a negative control outcome because no direct biological association with baseline magnesium status was expected.

### 2.7 Sensitivity, subgroup, and multivariable analyses

Three prespecified sensitivity analyses were conducted. The first analysis was restricted to patients without documented history of chronic kidney disease. Although patients with stage 4–5 chronic kidney disease or end-stage renal disease were excluded from the primary cohort, this sensitivity analysis further excluded any documented chronic kidney disease to evaluate whether the observed association was influenced by milder renal dysfunction. The second excluded patients who died during follow-up to evaluate the influence of differential mortality on the results. Third, the analysis was limited to patients with at least one recorded healthcare encounter during follow-up to reduce potential bias from incomplete outcome capture. Subgroup analyses were conducted according to sex and age group. Patients were stratified into male and female subgroups and into age categories of 50–65 and >65 years. Sequential multivariable Cox models were fitted with cohort membership as the main exposure. These models were progressively adjusted for demographics, vascular comorbidities, metabolic and nutritional conditions, lifestyle and systemic comorbidities, neuropsychiatric and cancer-related conditions, and medication use.

### 2.8 Association between high magnesium status and dementia risk

An exploratory analysis was performed to assess whether the risk of dementia was associated with high magnesium status, consistent with a potential non-linear or U-shaped relationship. As elevated magnesium levels may reflect impaired renal excretion, patients with a documented history of chronic kidney disease were excluded from this analysis. The high magnesium cohort included patients with two magnesium values >2.20 mg/dL, whereas the comparison cohort included patients with two values between 1.70 and 2.20 mg/dL. The second qualifying magnesium measurement was used as the index. The same 1-year landmark design, propensity score matching strategy, outcome definitions, and time-to-event frameworks were applied.

### 2.9 Statistical analysis

Time-to-event outcomes were analyzed using Cox proportional hazards models, and the results are reported as hazard ratios (HRs) with 95% confidence intervals (CIs). The primary analysis used a cause-specific modeling framework because the objective was to estimate the association between magnesium status and subsequent dementia occurrence rather than to model the absolute cumulative incidence in the presence of competing mortality. Because Fine–Gray subdistribution hazard modeling was not available within the TriNetX analytic module, an Aalen–Johansen competing risk analysis was performed descriptively in the unmatched cohort to estimate the cumulative incidence of all-cause dementia while treating mortality as a competing event. No formal statistical comparison was conducted for this analysis; therefore, the results were presented only as supplementary descriptive information. The proportional hazards assumption was examined using Schoenfeld residuals. Kaplan–Meier curves were generated to display dementia-free survival, and group differences were evaluated using log-rank tests.

For the primary outcome, E-values were calculated to estimate the minimum strength of association that an unmeasured confounder would need to have with both magnesium status and dementia to fully explain the observed association. Statistical significance for the primary analysis was defined as a two-sided *α* of 0.05. Analyses of secondary outcomes, control outcomes, subgroup models, sensitivity analyses, and high-magnesium analyses were considered exploratory; therefore, no multiplicity adjustment was applied. Missing data were handled using the available information in the database, and no imputation was performed.

## 3 Results

### 3.1 Patient selection and baseline characteristics

**Figure 1:**
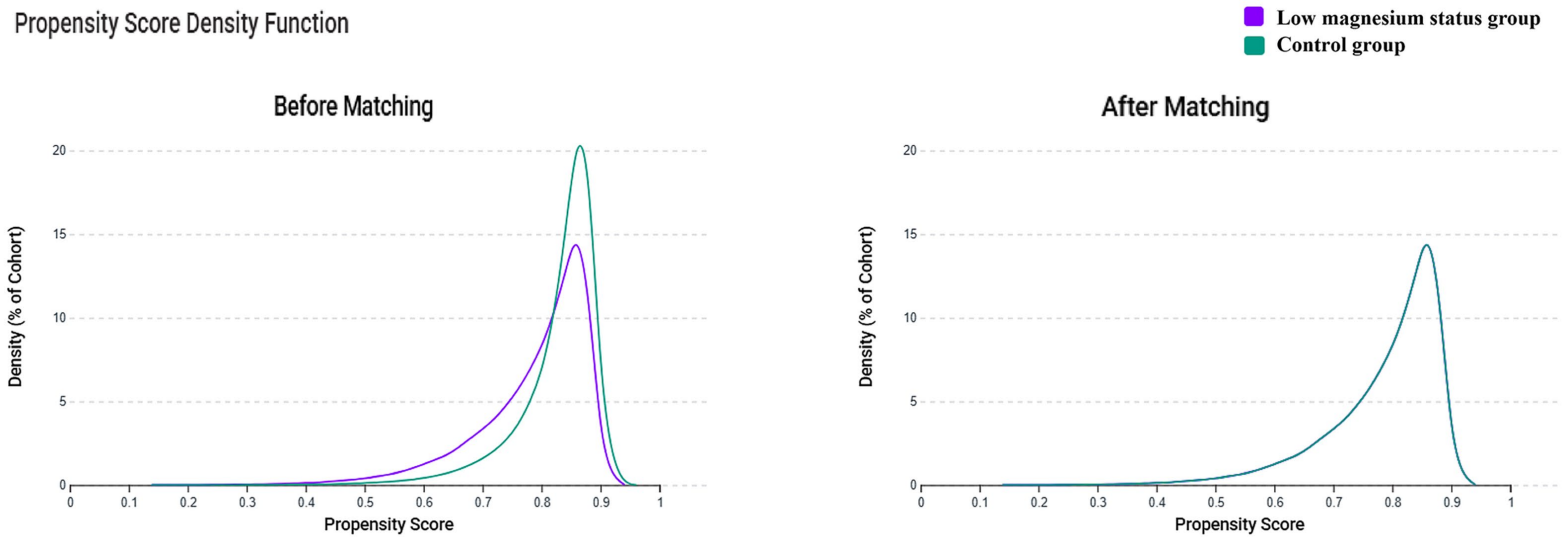
Propensity score density distributions before and after matching. Propensity score density plots comparing patients with low magnesium status and matched controls before and after propensity score matching. Before matching, the distributions showed incomplete overlap between groups, indicating baseline differences in measured covariates. After 1:1 propensity score matching, the distributions demonstrated improved overlap, supporting adequate covariate balance between the low magnesium status and control cohorts.

Before propensity score matching, 162,945 patients were included in the low magnesium status group and 801,069 patients were included in the control group (Table 1). After 1:1 propensity score matching, 162,936 patients remained in each group. The mean age after matching was 65.2 ± 11.7 years in the low magnesium status group and 65.4 ± 11.8 years in the control group. Women accounted for 54.4 and 54.6% of the low magnesium status and control groups, respectively. After matching, most baseline characteristics were well-balanced, with standardized mean differences <0.10 for demographic characteristics, comorbidities, medication use, and most laboratory variables. Propensity score density plots showed improved overlap between the groups after matching (Figure 1).

**Table 1:** Baseline characteristics of general population with low magnesium status and matched controls before and after propensity score matching.

Variables	Before matching		After matching			
	Low Mg group (*n* = 162,945)	Control group (*n* = 801,069)	SMD	Low Mg group (*n* = 162,936)	Control group (*n* = 162,936)	SMD
Patient characteristics						
Age at index (years)	64.9 ± 12.0	65.2 ± 11.7	0.031	65.2 ± 11.7	65.4 ± 11.8	0.013
BMI ≥ 30 (kg/m^2^)	45,091 (27.7)	207,375 (25.9)	0.040	45,089 (27.7)	44,198 (27.1)	0.012
Female	88,626 (54.4)	402,439 (50.2)	0.083	88,623 (54.4)	88,967 (54.6)	0.004
White	102,569 (62.9)	520,630 (65.0)	0.043	102,566 (62.9)	101,328 (62.2)	0.016
Black or African American	22,502 (13.8)	93,086 (11.6)	0.066	22,497 (13.8)	22,543 (13.8)	0.001
Asian	3,751 (2.3)	26,059 (3.3)	0.058	3,751 (2.3)	3,608 (2.2)	0.006
Comorbidities and Healthcare Utilization						
Factors influencing health status and contact with health services	89,184 (54.7)	389,389 (48.6)	0.123	89,175 (54.7)	86,944 (53.4)	0.027
Essential (primary) hypertension	64,789 (39.8)	271,244 (33.9)	0.123	64,784 (39.8)	62,897 (38.6)	0.024
Neoplasms	57,507 (35.3)	212,873 (26.6)	0.189	57,498 (35.3)	57,895 (35.5)	0.005
Diabetes mellitus	38,455 (23.6)	117,030 (14.6)	0.230	38,448 (23.6)	38,090 (23.4)	0.005
Ischemic heart diseases	24,486 (15.0)	118,432 (14.8)	0.007	24,485 (15.0)	23,059 (14.2)	0.025
Anxiety, dissociative, stress-related, somatoform and other nonpsychotic mental disorders	19,477 (12.0)	77,656 (9.7)	0.073	19,476 (12.0)	18,849 (11.6)	0.012
Overweight and obesity	19,025 (11.7)	83,899 (10.5)	0.038	19,024 (11.7)	18,095 (11.1)	0.018
Disorders of thyroid gland	18,548 (11.4)	80,874 (10.1)	0.042	18,548 (11.4)	17,880 (11.0)	0.013
Nicotine dependence	18,058 (11.1)	67,240 (8.4)	0.091	18,053 (11.1)	17,386 (10.7)	0.013
Diseases of liver	17,339 (10.6)	48,152 (6.0)	0.168	17,332 (10.6)	16,876 (10.4)	0.009
Sleep disorders	16,895 (10.4)	72,497 (9.1)	0.045	16,894 (10.4)	16,116 (9.9)	0.016
Mood disorders	16,687 (10.2)	63,538 (7.9)	0.080	16,686 (10.2)	16,183 (9.9)	0.010
Atrial fibrillation and flutter	13,520 (8.3)	61,844 (7.7)	0.021	13,520 (8.3)	12,886 (7.9)	0.014
COPD	13,027 (8.0)	45,813 (5.7)	0.090	13,026 (8.0)	12,586 (7.7)	0.010
Heart failure	12,969 (8.0)	51,951 (6.5)	0.057	12,967 (8.0)	12,565 (7.7)	0.009
Chronic kidney disease	10,774 (6.6)	35,846 (4.5)	0.094	10,770 (6.6)	10,577 (6.5)	0.005
Vitamin D deficiency	10,573 (6.5)	48,009 (6.0)	0.020	10,573 (6.5)	10,109 (6.2)	0.012
Alcohol related disorders	9,631 (5.9)	22,891 (2.9)	0.150	9,623 (5.9)	9,234 (5.7)	0.010
Malnutrition	7,870 (4.8)	15,347 (1.9)	0.162	7,863 (4.8)	7,220 (4.4)	0.019
Cerebrovascular diseases	5,854 (3.6)	25,689 (3.2)	0.021	5,853 (3.6)	5,589 (3.4)	0.009
Systemic connective tissue disorders	2,569 (1.6)	10,386 (1.3)	0.024	2,569 (1.6)	2,426 (1.5)	0.007
Laboratory data						
Hemoglobin ≥ 12 g/dL	95,845 (58.8)	437,719 (54.6)	0.084	95,838 (58.8)	96,431 (59.2)	0.007
Albumin ≥ 3.5 g/dL	81,103 (49.8)	336,776 (42.0)	0.156	81,095 (49.8)	81,607 (50.1)	0.006
HbA1c ≥ 9%	8,149 (5.0)	19,705 (2.5)	0.134	8,146 (5.0)	8,108 (5.0)	0.001
eGFR ≥60 mL/min/1.73 m^2^	105,254 (64.6)	456,803 (57.0)	0.156	105,245 (64.6)	106,078 (65.1)	0.011
C-reactive protein ≥ 10 mg/L	20,642 (12.7)	56,502 (7.1)	0.189	20,635 (12.7)	20,805 (12.8)	0.003
Vitamin B12 300–900 pg./mL	15,371 (9.4)	53,933 (6.7)	0.099	15,366 (9.4)	14,955 (9.2)	0.009
Medications						
Central nervous system medications	107,986 (66.3)	463,654 (57.9)	0.174	107,977 (66.3)	106,182 (65.2)	0.023
Benzodiazepine	59,687 (36.6)	225,337 (28.1)	0.182	59,681 (36.6)	58,858 (36.1)	0.010
Proton pump inhibitors	54,184 (33.3)	178,617 (22.3)	0.246	54,177 (33.3)	53,720 (33.0)	0.006
Diuretics	46,427 (28.5)	157,503 (19.7)	0.208	46,421 (28.5)	45,278 (27.8)	0.016
Magnesium	36,016 (22.1)	119,931 (15.0)	0.184	36,010 (22.1)	35,620 (21.9)	0.006
Insulins and analogues	35,346 (21.7)	99,025 (12.4)	0.250	35,337 (21.7)	35,136 (21.6)	0.003
Anticonvulsants	25,677 (15.8)	92,436 (11.5)	0.123	25,670 (15.8)	25,126 (15.4)	0.009
Blood glucose lowering drugs, excl. Insulins	23,711 (14.6)	66,713 (8.3)	0.196	23,705 (14.5)	23,478 (14.4)	0.004
Anticholinergics	15,835 (9.7)	54,751 (6.8)	0.105	15,834 (9.7)	15,608 (9.6)	0.005

Data are presented as *n* (%) or mean ± standard deviation. BMI, body mass index; COPD, chronic obstructive pulmonary disease; eGFR, estimated glomerular filtration rate; HbA1c, hemoglobin A1c; Mg, magnesium; SMD, standardized mean difference.

### 3.2 Primary and secondary outcomes

**Figure 2:**
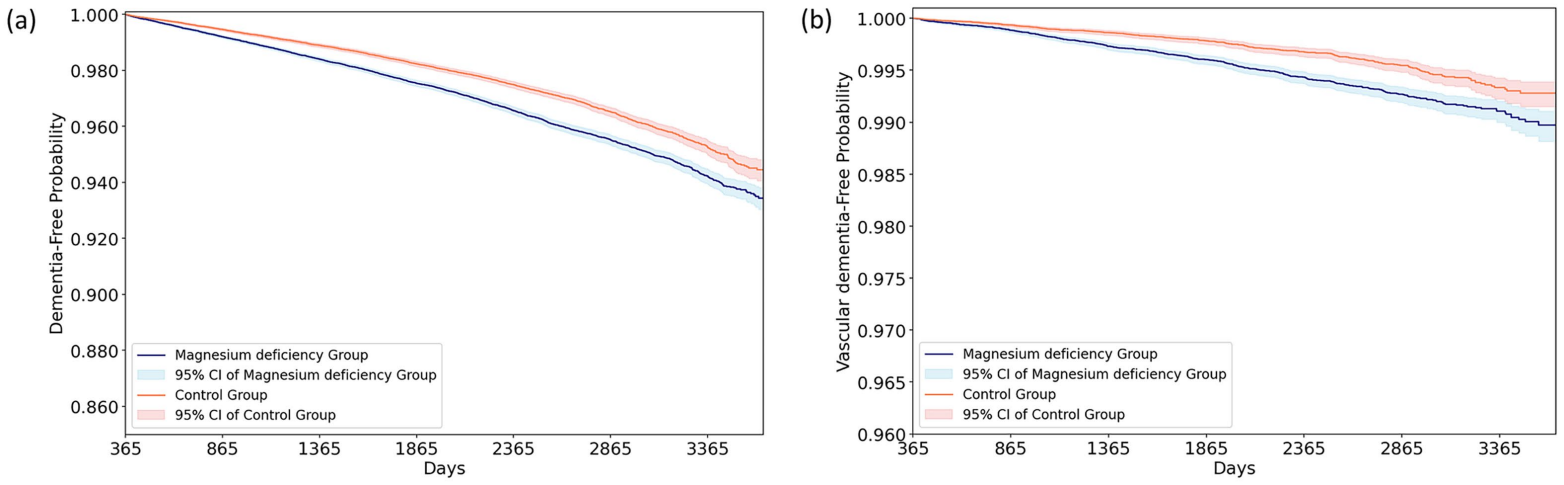
Kaplan–Meier curves for dementia-free survival after propensity score matching. Kaplan–Meier curves comparing dementia-free survival between patients with low magnesium status and matched controls for **(a)** all-cause dementia and **(b)** Vascular dementia. Low magnesium status was associated with a higher risk of incident all-cause dementia (HR, 1.33; 95% CI, 1.26–1.40; *p* < 0.001) and vascular dementia (HR, 1.63; 95% CI, 1.42–1.88; *p* < 0.001) during the 10 year follow-up period after the 1 year landmark period. HR, hazard ratio; CI, confidence interval. TriNetX exported survival probability estimates with 95% confidence intervals but did not provide the number of patients at risk at selected time points; therefore, number-at-risk tables could not be generated.

During the 10-year observation window after the 1-year landmark period, incident all-cause dementia occurred in 1.91% of patients in the low magnesium status group and 1.49% of patients in the control group (HR, 1.33; 95% CI, 1.26–1.40; *p* < 0.001) (Table 2). The corresponding E-value was 1.99 for the point estimate and 1.83 for the lower confidence interval bound. Kaplan–Meier analysis showed a significant difference in dementia-free survival between the two groups (Figure 2a). For dementia subtypes, low magnesium status was associated with Alzheimer’s disease (HR 1.24, *p* < 0.001), vascular dementia (HR 1.63, *p* < 0.001) (Figure 2b), and other or unspecified dementia (HR 1.31, *p* < 0.001). Low magnesium status was also associated with stroke (HR 1.37, *p* < 0.001) and all-cause mortality (HR 1.52, *p* < 0.001). In the prespecified control outcome analyses, low magnesium status was associated with hypokalemia (HR 1.44, *p* < 0.001), whereas no association was observed with acute appendicitis (HR 1.02, *p* = 0.782).

**Table 2:** Association between low magnesium status and 10-year risk of incident dementia after propensity score matching.

Outcome	LMS group (*n* = 162,936)	Control group (*n* = 162,936)	HR (95% CI)	*p*-value
	Events (%)	Events (%)		
Primary outcome				
All-cause dementia	3,116 (1.91)	2,430 (1.49)	1.33 (1.26–1.40)	<0.001
Secondary outcomes				
Alzheimer’s disease	789 (0.48)	658 (0.40)	1.24 (1.12–1.37)	<0.001
Vascular dementia	500 (0.31)	317 (0.19)	1.63 (1.42–1.88)	<0.001
Other type of dementia	2,816 (1.73)	2,218 (1.36)	1.31 (1.24–1.39)	<0.001
Stroke	5,503 (3.38)	4,177 (2.56)	1.37 (1.32–1.43)	<0.001
Mortality	18,075 (11.09)	12,389 (7.60)	1.52 (1.48–1.55)	<0.001
Positive control outcome				
Hypokalemia	38,216 (23.46)	28,921 (17.75)	1.44 (1.42–1.46)	<0.001
Negative control outcome				
Appendicitis	313 (0.19)	316 (0.19)	1.02 (0.87–1.20)	0.782

LMS, low magnesium status; HR, hazard ratio; CI, confidence interval.

### 3.3 Sensitivity analyses and subgroup analyses

The association between low magnesium status and incident all-cause dementia remained consistent across the three sensitivity analyses (Table 3). In the analysis restricted to patients without any history of chronic kidney disease, low magnesium status was associated with incident all-cause dementia (HR 1.28, *p* < 0.001). Similar associations were observed after excluding patients who died during follow-up (HR 1.27, *p* < 0.001) and after restricting the cohort to patients with at least one healthcare visit during follow-up (HR 1.29, *p* < 0.001).

**Table 3:** Sensitivity analyses of the association between low magnesium status and 10-year risk of incident dementia.

Outcomes	Model I (*n* = 139,486 for each group)		Model II (*n* = 137,723 for each group)		Model III (*n* = 107,871 for each group)	
	HR (95% CI)	*p*-value	HR (95% CI)	*p*-value	HR (95% CI)	*p*-value
All-cause dementia	1.28 (1.20–1.36)	<0.001	1.27 (1.19–1.36)	<0.001	1.29 (1.22–1.37)	<0.001
Alzheimer’s disease	1.10 (0.98–1.23)	0.115	1.17 (1.04–1.32)	0.011	1.19 (1.07–1.33)	0.002
Vascular dementia	1.41 (1.21–1.65)	<0.001	1.47 (1.25–1.74)	<0.001	1.41 (1.22–1.63)	<0.001
Other type of dementia	1.28 (1.20–1.36)	<0.001	1.26 (1.18–1.35)	<0.001	1.29 (1.22–1.37)	<0.001
Stroke	1.35 (1.30–1.42)	<0.001	1.34 (1.28–1.41)	<0.001	1.35 (1.29–1.40)	<0.001
Mortality	1.52 (1.48–1.56)	<0.001	—	—	1.63 (1.59–1.68)	<0.001

Model I was restricted to patients without a history of CKD. Model II excluded patients who died during the follow-up period. Model III was restricted to patients who have at least one health visit during follow-up.

In sex-stratified analyses (Table 4), low magnesium status was associated with all-cause dementia in both men (HR 1.27, *p* < 0.001) and women (HR 1.26, *p* < 0.001), with no evidence of an interaction by sex (*p* for interaction = 0.894). In age-stratified analyses (Table 5), the association was observed among patients aged 50–65 years (HR 1.45, *p* = 0.004) and those aged >65 years (HR 1.35, *p* < 0.001), with no evidence of interaction by age for all-cause dementia (*p* for interaction = 0.608).

**Table 4:** Sex-stratified subgroup analyses of the association between low magnesium status and dementia risk.

Outcomes	Male (*n* = 61,673)		Female (*n* = 79,562)		P for interaction
	HR (95% CI)	*p*-value	HR (95% CI)	*p*-value	
All-cause dementia	1.27 (1.15–1.39)	<0.001	1.26 (1.18–1.35)	<0.001	0.894
Alzheimer’s disease	1.10 (0.90–1.33)	0.355	1.23 (1.08–1.40)	0.002	0.342
Vascular dementia	1.33 (1.05–1.67)	0.016	1.49 (1.24–1.79)	<0.001	0.449
Other type of dementia	1.29 (1.17–1.43)	<0.001	1.27 (1.18–1.36)	<0.001	0.804
Stroke	1.40 (1.31–1.50)	<0.001	1.34 (1.27–1.42)	<0.001	0.331
Mortality	1.49 (1.44–1.55)	<0.001	1.44 (1.39–1.49)	<0.001	0.187

HR, hazard ratio; CI, confidence interval.

**Table 5:** Age-stratified subgroup analyses of the association between low magnesium status and dementia risk.

Outcomes	50–65 years (*n* = 46,289)		>65 years (*n* = 105,788)		P for interaction
	HR (95% CI)	*p*-value	HR (95% CI)	*p*-value	
All-cause dementia	1.45 (1.13–1.88)	0.004	1.35 (1.28–1.43)	<0.001	0.608
Alzheimer’s disease	0.85 (0.44–1.65)	0.630	1.22 (1.09–1.36)	<0.001	0.242
Vascular dementia	1.62 (0.90–2.92)	0.105	1.57 (1.35–1.82)	<0.001	0.925
Other type of dementia	1.32 (1.01–1.75)	0.045	1.36 (1.28–1.44)	<0.001	0.836
Stroke	1.37 (1.25–1.50)	<0.001	1.41 (1.34–1.48)	<0.001	0.584
Mortality	1.78 (1.67–1.89)	<0.001	1.52 (1.48–1.56)	<0.001	<0.001

HR, hazard ratio; CI, confidence interval.

### 3.4 Sequential multivariable cox regression

In sequential multivariable Cox models (Table 6), the association between low magnesium status and incident all-cause dementia remained significant across all levels of adjustment. The unadjusted model showed an HR of 1.33 (*p* < 0.001). After adjusting for demographics, the HR was 1.34 (*p* < 0.001). Further adjustment for vascular comorbidities yielded an HR of 1.36 (*p* < 0.001). Additional adjustments for metabolic and nutritional factors, lifestyle and systemic comorbidities, neuropsychiatric and cancer-related conditions, and medication use yielded HRs ranging from 1.30 to 1.32, all with *p* < 0.001.

**Table 6:** Sequential multivariable cox regression models evaluating the association between low magnesium status and incident dementia.

Model	Matching/adjustment variables included	HR (95% CI)	*p*-value
Model I	Unadjusted model	1.33 (1.26–1.40)	<0.001
Model II	Demographics (sex, age at index, race/ethnicity)	1.34 (1.29–1.40)	<0.001
Model III	Model II + vascular comorbidities (essential hypertension, ischemic heart disease, atrial fibrillation/flutter, cerebrovascular diseases)	1.36 (1.31–1.41)	<0.001
Model IV	Model III + metabolic and nutritional factors (diabetes mellitus, overweight/obesity, thyroid disorders, vitamin D deficiency, malnutrition)	1.32 (1.27–1.37)	<0.001
Model V	Model IV + lifestyle/systemic comorbidities (nicotine dependence, liver disease, chronic kidney disease, alcohol-related disorders)	1.30 (1.26–1.35)	<0.001
Model VI	Model V + neuropsychiatric and cancer-related conditions (anxiety/dissociative/stress-related/somatoform disorders, neoplasms, sleep disorders, mood disorders)	1.32 (1.27–1.37)	<0.001
Model VII	Model VI + medications (benzodiazepine derivative sedatives/hypnotics, proton pump inhibitors, diuretics, anticonvulsants, insulins and analogues, anticholinergics)	1.32 (1.28–1.38)	<0.001

### 3.5 Aalen–Johansen competing risk analysis

In the descriptive Aalen–Johansen competing risk analysis of the unmatched cohort, the cumulative incidence of all-cause dementia was higher in the low magnesium status group than in the normal magnesium status group (5.9% vs. 5.1%) when mortality was treated as a competing event (Table 7). The cumulative incidence of mortality was also higher in the low magnesium status group than in the normal magnesium status group (27.5% vs. 18.1%). Because no formal statistical comparison was conducted for this analysis, these findings were interpreted as supplementary descriptive information.

**Table 7:** Competing risk analysis of all-cause dementia and mortality by magnesium status.

Magnesium status	Outcome	% of cohort	Cumulative incidence at end of time window
Low magnesium status	Mortality	10.4%	27.5%
Low magnesium status	All-cause dementia	2.0%	5.9%
Normal magnesium status	Mortality	6.2%	18.1%
Normal magnesium status	All-cause dementia	1.5%	5.1%

Values were derived from the Aalen–Johansen competing risk analysis in the unmatched cohort. The cumulative incidence of both mortality and all-cause dementia was higher in the low magnesium status group than in the normal magnesium status group. Low magnesium status was defined as two magnesium values <1.70 mg/dL, and normal magnesium status was defined as two values between 1.70 and 2.20 mg/dL.

### 3.6 Association between high magnesium status and dementia risk

In the exploratory analysis restricted to individuals without documented chronic kidney disease, 168,243 patients were included in each group after matching (Table 8). High magnesium status was associated with a higher risk of incident all-cause dementia (HR 1.25, *p* < 0.001). Similar associations were observed for vascular dementia (HR 1.40, *p* < 0.001), other or unspecified dementia (HR 1.25, *p* < 0.001), stroke (HR 1.28, *p* < 0.001), and mortality (HR 1.31, *p* < 0.001). The association with Alzheimer’s disease was not statistically significant (HR 1.09, *p* = 0.096).

**Table 8:** Association between high magnesium status and 10-year risk of incident dementia in individuals without documented CKD history.

Outcome	High Mg status group (*n* = 168,243)	Control group (*n* = 168,243)	HR (95% CI)	*p*-value
	Events (%)	Events (%)		
All-cause dementia	2,923 (1.74)	2,371 (1.41)	1.25 (1.19–1.32)	<0.001
Alzheimer’s disease	795 (0.47)	740 (0.44)	1.09 (0.99–1.20)	0.096
Vascular dementia	426 (0.25)	309 (0.18)	1.40 (1.21–1.62)	<0.001
Other type of dementia	2,644 (1.57)	2,158 (1.28)	1.25 (1.18–1.32)	<0.001
Stroke	5,089 (3.03)	4,080 (2.43)	1.28 (1.23–1.33)	<0.001
Mortality	14,490 (8.61)	11,312 (6.72)	1.31 (1.27–1.34)	<0.001

This exploratory analysis included adults without documented chronic kidney disease. High magnesium status was defined as two magnesium values >2.20 mg/dL. CI, confidence interval; CKD, chronic kidney disease; HR, hazard ratio; Mg, magnesium.

## 4 Discussion

In this large propensity score–matched cohort study, repeatedly low magnesium status was associated with a higher risk of incident all-cause dementia, corresponding to a 33% relative increase compared with normal magnesium status. Among the dementia subtypes, the association was most pronounced for vascular dementia. Low magnesium status was also associated with higher risks of stroke and all-cause mortality. Prespecified control outcome analyses were consistent with the expected pattern, showing an association with hypokalemia but not with acute appendicitis. The findings were generally stable across the sensitivity analyses, subgroup analyses, and sequential multivariable Cox models. In an exploratory analysis excluding patients with chronic kidney disease, repeatedly high magnesium levels showed a similar association pattern, raising the possibility of a nonlinear or U-shaped relationship between magnesium status and dementia risk.

Our results are generally aligned with prior cohort evidence on circulating magnesium and dementia risk while adding several methodological features (13, 15, 17). The Rotterdam Study reported a higher all-cause dementia risk at both low and high serum magnesium concentrations, whereas the ARIC-NCS found an association between low midlife serum magnesium and incident dementia over long-term follow-up (13–16). Similarly, the Copenhagen General Population Study showed that extreme plasma magnesium concentrations were associated with non-Alzheimer’s dementia (17), and a systematic review by Chen et al. summarized the evidence linking magnesium biomarkers and dietary magnesium intake with cognitive disorders (22). However, important differences distinguish this study from earlier work. Many previous studies classified magnesium exposure using a single baseline measurement, often from cohorts enrolled in earlier decades, which may not fully capture persistent magnesium status or reflect contemporary clinical practice.

Notably, previous studies have reported less consistent findings for Alzheimer’s disease than for all-cause or vascular dementia. One explanation may be that single-measurement exposure definitions are vulnerable to non-differential misclassification, which can bias estimates toward the null. Such misclassification may be more likely to obscure weaker associations, such as those involving Alzheimer’s disease, while leaving stronger vascular-related associations more apparent. In the present study, repeated magnesium measurements may have improved exposure specificity and allowed detection of a modest association with Alzheimer’s disease in the primary analysis. However, this association was attenuated after restricting the cohort to patients without chronic kidney disease, suggesting that the Alzheimer’s disease finding may partly reflect renal comorbidity burden or related systemic vulnerability. Whether this association is independent of vascular-mediated pathways requires further investigation.

Several biological pathways may link magnesium status to the risk of developing dementia. Magnesium plays a critical role in maintaining cerebrovascular integrity by modulating endothelial function, vascular smooth muscle tone, and arterial calcification (23–27). Magnesium deficiency is associated with endothelial dysfunction, increased vascular stiffness, and impaired cerebral blood flow autoregulation, all of which may contribute to chronic cerebral hypoperfusion and small vessel disease (28, 29). These cerebrovascular mechanisms are consistent with our observation that vascular dementia exhibited the strongest association with low magnesium status in all analyses. Additionally, magnesium modulates N-methyl-D-aspartate (NMDA) receptor activity, and sustained magnesium depletion may alter excitatory neurotransmission and promote excitotoxic neuronal injuries (30, 31). Low magnesium levels have also been implicated in systemic inflammation and insulin resistance (32), both of which are recognized contributors to neurodegeneration. Importantly, these mechanisms are not mutually exclusive and may operate through overlapping neurovascular and neuroinflammatory pathways of action.

The observation that vascular dementia was the predominant subtype associated with both low and high magnesium statuses merits a specific discussion. In the primary analysis, the HR for vascular dementia (1.63) was notably higher than that for Alzheimer’s disease (1.24) or other dementia subtypes (1.31). A parallel pattern was observed in the high magnesium analysis, where vascular dementia (HR 1.40) and stroke (HR 1.28) remained significantly associated, whereas Alzheimer’s disease did not reach statistical significance (HR 1.09, *p* = 0.096). These findings suggest that the association between magnesium and dementia may be substantially mediated through cerebrovascular pathways rather than through primary neurodegenerative processes. The concurrent association with stroke further supports this hypothesis. The attenuation of the Alzheimer’s disease association after excluding patients with chronic kidney disease in the sensitivity analysis (HR 1.10, *p* = 0.115) raises the possibility that some of the observed Alzheimer’s disease signal in the primary analysis may have been partially influenced by renal comorbidity burden rather than by magnesium status per se.

The subgroup findings should be interpreted cautiously. Low magnesium status was associated with overall dementia in both men and women and in both age strata, and the interaction tests for overall dementia were not significant. Therefore, these results do not provide strong evidence of effect modification by sex or age. The numerically higher HR in patients aged 50–65 years may reflect a lower baseline dementia risk in this younger group, making relative differences more apparent, but the smaller number of events may also reduce precision. Similarly, apparent sex-related differences in dementia subtypes may reflect differences in baseline risk, comorbidity profiles, diagnostic patterns, or statistical variability rather than true biological differences.

This study had several methodological strengths. First, propensity score matching incorporated a broad set of covariates, including demographic and cardiometabolic factors, medications affecting magnesium homeostasis, and laboratory indicators of renal function, glycemic control, inflammation, nutrition, and hematologic status (33–35). Conditions related to both magnesium depletion and cognitive risk, such as alcohol-related disorders, malnutrition, liver disease, and nicotine dependence, were also included (36, 37). The association was further supported by an E-value of 1.99 for the point estimate and 1.83 for the lower confidence interval bound, although this should be interpreted only as a sensitivity measure and not as evidence of causality. Second, requiring two qualifying magnesium measurements reduced misclassification from transient electrolyte abnormalities. Third, the 1-year landmark design reduced potential reverse causation. Fourth, positive and negative control outcomes, sequential multivariable Cox models, and the large matched sample strengthened the robustness of the findings. Finally, the exploratory high-magnesium analysis provided preliminary evidence supporting a possible U-shaped relationship (38).

Several limitations should be acknowledged. First, as an electronic health record–based study, dementia ascertainment relied on ICD-10-CM diagnostic codes, which may have underestimated the true incidence and introduced misclassification, particularly for dementia subtypes. The absence of neuroimaging or biomarker confirmation means that subtype classifications should be interpreted with caution. In addition, dementia coding practices may vary across institution types within the TriNetX network, including hospitals, outpatient clinics, and primary care settings. Such variation may have influenced the relative frequency of dementia subtypes and should be considered when interpreting subtype-specific findings. Second, despite comprehensive propensity score matching and sequential covariate adjustment, residual confounding by unmeasured variables cannot be excluded from the study. Dietary magnesium intake, supplemental magnesium use, and ionized magnesium concentrations were not available in the database, and these factors may have influenced the observed associations. In addition, TriNetX allows cohort identification based on the occurrence of at least two magnesium measurements within a prespecified assessment window but does not provide the exact interval between the two qualifying measurements within the analytic module. Therefore, we could not report the median time between magnesium measurements or distinguish short-interval repeated testing from longer-term repeated assessment. This limitation may have introduced heterogeneity in the clinical meaning of repeated magnesium status. Although two magnesium measurements within 1 year were required to reduce misclassification from a single transient value, serum magnesium may still reflect recent nutritional, clinical, or treatment-related status rather than long-term magnesium homeostasis. Third, the included patients were distributed across multiple US regions and non-US healthcare organizations; however, TriNetX reports geographic distribution based on healthcare-organization headquarters rather than patient residence or laboratory site. Thus, interinstitutional differences in laboratory platforms and reference practices may have introduced exposure misclassification and may limit generalizability. Fourth, although a cause-specific modeling framework was used, the competing risk of death remains a consideration in this study. The Aalen–Johansen analysis showed a higher cumulative incidence of all-cause dementia in the low magnesium status group despite higher competing mortality, supporting the direction of the primary findings. However, because formal Fine–Gray subdistribution modeling was not available in TriNetX, this analysis should be interpreted as descriptive rather than confirmatory. Fifth, although the repeated measurement requirement and landmark design were intended to strengthen causal inference, this study remains observational, and the findings should be interpreted as identifying associations rather than establishing causal relationships between variables.

## 5 Conclusion

In this large contemporary propensity score–matched cohort study, repeatedly documented low magnesium status was associated with an increased risk of incident all-cause dementia, with the strongest association observed for vascular dementia. An exploratory analysis of high magnesium status revealed a similar pattern, supporting a potential U-shaped relationship. These findings highlight the potential relevance of magnesium homeostasis to long-term cognitive outcomes and may inform future research on modifiable metabolic risk factors for the prevention of dementia.

## Data Availability

The raw data supporting the conclusions of this article will be made available by the authors, without undue reservation.
