# Supplementary material for: Association between low magnesium status and new-onset dementia in the general population: a propensity score-matched cohort study
Source: Front Nutr. 2026 Jun 8;13:1886820. doi: 10.3389/fnut.2026.1886820 (PMC13283792; doi:10.3389/fnut.2026.1886820)
Supplement: Supplementary file 1 [file Table_1.DOCX]

**Supplemental Table 1.** Codes Used for Cohort Definition, Inclusion/Exclusion Criteria, Outcome Definitions, and Variables for Propensity Score Matching

| Category | Variable / concept | Definition / codes |
| --- | --- | --- |
| Inclusion criteria | Age | ≥50 years |
|  | Low magnesium status | Two serum/plasma magnesium values <1.7 mg/dL within 1 year; LOINC 19123-9 |
|  | Control magnesium status | Two serum/plasma magnesium values 1.70–2.20 mg/dL within 1 year; LOINC 19123-9 |
|  | Index date | Date of the second qualifying magnesium measurement |
|  | Study period | Magnesium measurements recorded between January 1, 2016, and December 31, 2023 |
| Landmark design | Landmark period | Outcome follow-up began 365 days after index date and continued to 3650 days after index date |
|  | Dementia before landmark | Excluded if dementia was recorded before or within 365 days after index date |
|  | Death before landmark | Excluded if deceased or mortality code recorded within 365 days after index date |
| Exclusion criteria | Acute kidney failure | ICD-10-CM: N17 |
|  | Sepsis | ICD-10-CM: A41 |
|  | Severe sepsis | ICD-10-CM: R65.2 |
|  | Critical care services | CPT: 1013729 |
|  | Dementia before index / landmark | ICD-10-CM: F01, F02, F03, G30–G32 |
|  | Advanced chronic kidney disease | ICD-10-CM: N18.4, N18.5, N18.6 |
|  | Dialysis dependence | ICD-10-CM: Z99.2 |
|  | Bipolar disorder | ICD-10-CM: F31 |
|  | Schizophrenia-spectrum and other psychotic disorders | ICD-10-CM: F20–F29 |
|  | Parkinson’s disease | ICD-10-CM: G20 |
|  | Bariatric surgery status | ICD-10-CM: Z98.84 |
|  | Bariatric surgery procedures | CPT: 1007385 |
|  | Cerebral infarction | ICD-10-CM: I63 |
|  | Nontraumatic intracerebral hemorrhage | ICD-10-CM: I61 |
|  | Transient ischemic attack | ICD-10-CM: G45 |
|  | Central nervous system malignancy | ICD-10-CM: C70, C71, C72 |
|  | Benign CNS neoplasm | ICD-10-CM: D32, D33 |
|  | Neoplasm of uncertain/unspecified behavior of CNS | ICD-10-CM: D42, D49.6 |
|  | Intracranial injury | ICD-10-CM: S06 |
|  | COVID-19 | ICD-10-CM: U07.1; SARS-CoV-2 RNA test: TNX 9088 |
| Primary outcome | Overall dementia | ICD-10-CM: F01, F02, F03, G30 |
| Secondary outcomes | Alzheimer’s disease | ICD-10-CM: G30 |
|  | Vascular dementia | ICD-10-CM: F01 |
|  | Other or unspecified dementia | ICD-10-CM: F02, F03 |
|  | Stroke | ICD-10-CM: I63, G45 |
|  | All-cause mortality | Deceased status; ICD-10-CM: R99 |
| Control outcomes | Hypokalemia | ICD-10-CM: E87.6; serum/plasma/blood potassium ≤3.5 mmol/L, TNX 9028 |
|  | Acute appendicitis | ICD-10-CM: K35 |
| Propensity-score matching variables: demographics | Age at index | Age at index |
|  | Sex | Female; male as reference |
|  | Race/ethnicity | White: 2106-3; Black or African American: 2054-5; Asian: 2028-9 |
| Propensity-score matching variables: comorbidities | Essential hypertension | ICD-10-CM: I10 |
|  | Diabetes mellitus | ICD-10-CM: E08–E13 |
|  | Overweight and obesity | ICD-10-CM: E66 |
|  | Nicotine dependence | ICD-10-CM: F17 |
|  | Ischemic heart diseases | ICD-10-CM: I20–I25 |
|  | Chronic kidney disease | ICD-10-CM: N18 |
|  | Alcohol-related disorders | ICD-10-CM: F10 |
|  | Cerebrovascular diseases | ICD-10-CM: I60–I69 |
|  | Chronic obstructive pulmonary disease | ICD-10-CM: J44 |
|  | Malnutrition | ICD-10-CM: E40–E46 |
|  | Healthcare utilization / contact with health services | ICD-10-CM: Z00–Z99 |
|  | Sleep disorders | ICD-10-CM: G47 |
|  | Heart failure | ICD-10-CM: I50 |
|  | Atrial fibrillation and flutter | ICD-10-CM: I48 |
|  | Systemic connective tissue disorders | ICD-10-CM: M30–M36 |
|  | Mood disorders | ICD-10-CM: F30–F39 |
|  | Anxiety, dissociative, stress-related, somatoform, and other nonpsychotic mental disorders | ICD-10-CM: F40–F48 |
|  | Diseases of liver | ICD-10-CM: K70–K77 |
|  | Thyroid disorders | ICD-10-CM: E00–E07 |
|  | Vitamin D deficiency | ICD-10-CM: E55 |
|  | Neoplasms | ICD-10-CM: C00–D49 |
| Propensity-score matching variables: medications | Benzodiazepine derivative sedatives/hypnotics | RxNorm category: CN302 |
|  | Insulins and analogues | RxNorm category: A10A |
|  | Blood glucose–lowering drugs, excluding insulins | RxNorm category: A10B |
|  | Central nervous system medications | RxNorm category: CN000 |
|  | Diuretics | RxNorm category: CV700 |
|  | Anticholinergics | RxNorm category: S01FA |
|  | Anticonvulsants | RxNorm category: CN400 |
|  | Magnesium medications/supplements | RxNorm category: TN460 |
|  | Proton pump inhibitors | RxNorm category: A02BC |
| Propensity-score matching variables: laboratory data | Albumin | TNX 9045; albumin ≥3.5 g/dL |
|  | eGFR | LOINC 98979-8; eGFR ≥60 mL/min/1.73 m² |
|  | Hemoglobin A1c | TNX 9037; HbA1c ≥9% |
|  | C-reactive protein | TNX 9063; CRP ≥10 mg/L |
|  | Hemoglobin | TNX 9014; hemoglobin ≥12 g/dL |
|  | Body mass index | TNX 9083; BMI ≥30 kg/m² |
|  | Vitamin B12 | TNX 9065; vitamin B12 300–900 pg/mL |

**Supplemental Figure 1.** Flowchart of patient selection and cohort assembly.

Adults aged ≥50 years with at least two serum magnesium measurements recorded within a 1-year assessment window were identified from the TriNetX Global Collaborative Network (2016–2023). Because the TriNetX platform applies all inclusion and exclusion criteria simultaneously during cohort assembly rather than in a sequential stepwise manner, individual exclusion counts for each criterion could not be ascertained. Therefore, sample sizes are presented at the pre-matching and post-matching stages. PSM, propensity score matching; CKD, chronic kidney disease; ESRD, end-stage renal disease; CNS, central nervous system.

**
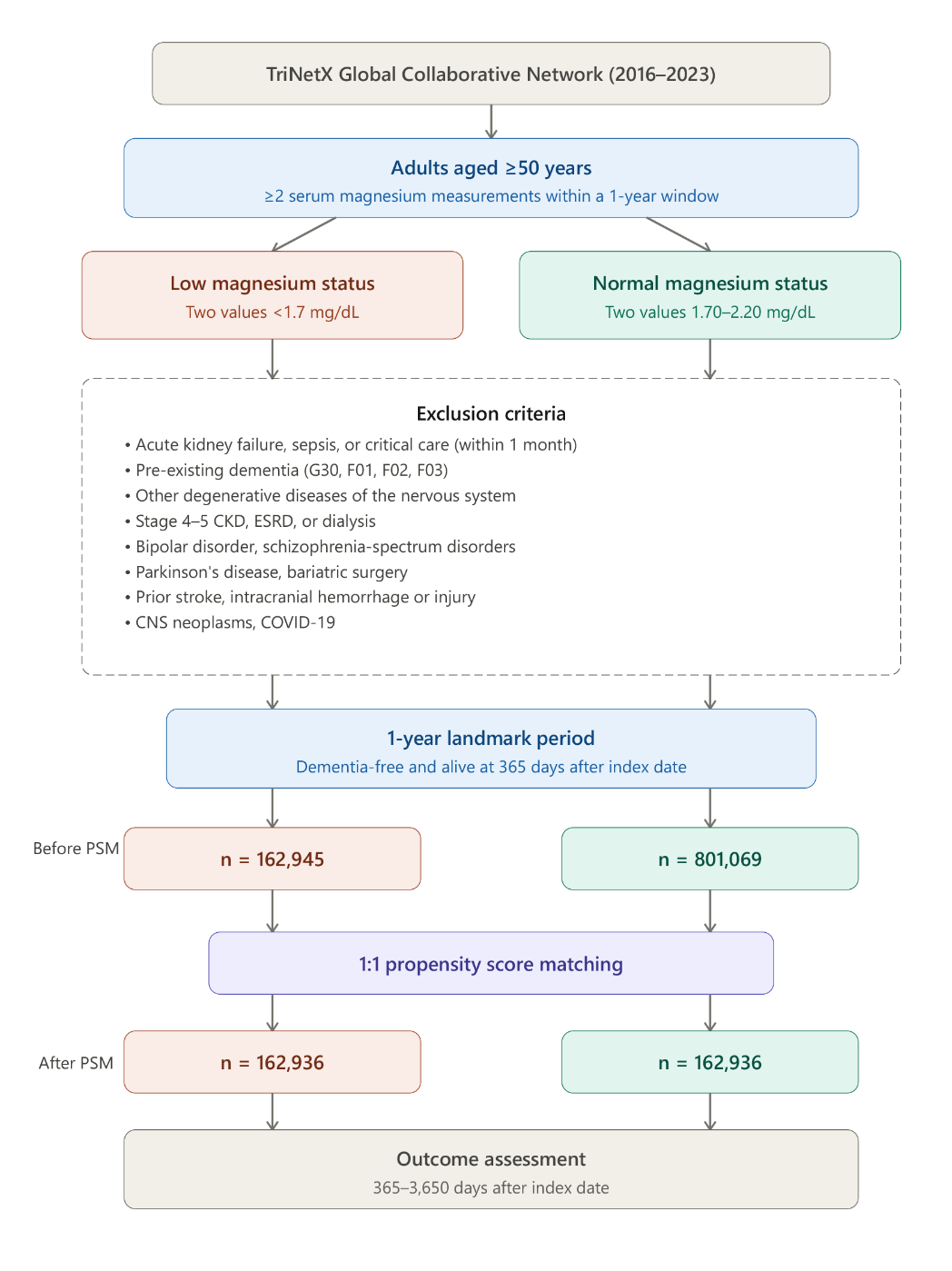
**
